# Clinical Relevance of Fluid Volume Status Assessment by Bioimpedance Spectroscopy in Children Receiving Maintenance Hemodialysis or Peritoneal Dialysis

**DOI:** 10.3390/jcm10010079

**Published:** 2020-12-28

**Authors:** Peong Gang Park, Jeesu Min, Seon Hee Lim, Ji Hyun Kim, Yo Han Ahn, Il-Soo Ha, Hee Gyung Kang

**Affiliations:** 1 Ministry of Health and Welfare, Sejong 339-012, Korea; pedpeong@gmail.com; 2 Department of Pediatrics, Seoul National University Children’s Hospital, Seoul 03080, Korea; jeesu.min@gmail.com (J.M.); kkrsh@naver.com (S.H.L.); ilsooha@snu.ac.kr (I.-S.H.); kanghg@snu.ac.kr (H.G.K.); 3 Department of Pediatrics, Seoul National University College of Medicine, Seoul 03080, Korea; pedkimji@gmail.com; 4 Department of Pediatrics, Seoul National University Bundang Hospital, Seongnam 13620, Korea; 5 Medical Research Center, Kidney Research Institute, Seoul National University College of Medicine, Seoul 03080, Korea; 6 Wide River Institute of Immunology, Seoul National University, Hongcheon 25159, Korea

**Keywords:** bioimpedance spectroscopy, kidney failure, hypertension

## Abstract

Bioimpedance spectroscopy (BIS) is a noninvasive method used to evaluate body fluid volume status in dialysis patients, but reports on its effectiveness in pediatrics are scarce. We investigated the correlation between BIS and clinical characteristics and identified the changes in patients whose dialysis prescription was modified based on BIS. The medical records of children on maintenance dialysis who had undergone BIS between 2017 and 2019 were reviewed. Of the 49 patients, 14 were overhydrated, based on the >15% proportion of overhydration relative to extracellular water (OH/ECW) measured by BIS. Intake of ≥two antihypertensive medications was noted in the majority (85.7%) of children with fluid overload and only in 48.6% of those without fluid overload (*p* = 0.017). Elevated blood pressure despite medication use was significantly more common in patients with fluid overload than in those without fluid overload (78.6% vs. 45.7%, *p* = 0.037). Of the 14 overhydrated children, 13 (92.9%) had significant changes in body weight, OH/ECW, the number of antihypertensive drugs, left ventricular end-diastolic diameter, and cardiothoracic ratio after the change in dialysis prescription. BIS is a useful and noninvasive method to assess fluid status in dialysis children. Long-term follow-up and correlation with a more objective clinical indicator of fluid overload is necessary to verify the clinical effectiveness of BIS.

## 1. Introduction

Achievement of adequate fluid balance is one of the most important treatment goals in children with kidney failure with replacement therapy (KFRT). Fluid overload from inadequate fluid removal during dialysis can lead to edema, hypertension, and cardiovascular morbidity. On the other hand, excessive fluid removal can cause volume depletion, which can lead to hypotension, cardiac stunning during dialysis, and accelerated loss of residual kidney function [1]. Inadequate fluid balance from fluid overload and volume depletion has been associated with increased morbidity and mortality in both adults and children [2,3,4,5,6]. However, determination of fluid status in patients on dialysis is often not straightforward. Usually, fluid status assessment is based on clinical symptoms and physical examination signs, such as hypertension, edema, interdialytic weigh gain, and intradialytic hypotension during hemodialysis (HD); however, these clinical features are often unreliable [7,8,9]. In children, fluid status assessment is even more challenging because of the continuous increase in lean body weight with growth. Therefore, more objective methods to assess fluid status are necessary; these include bioimpedance spectroscopy (BIS), inferior vena cava (IVC) parameters, relative blood volume monitoring, N-terminal pro-brain natriuretic peptide (NT-proBNP), and lung ultrasound [10].

Among the above-mentioned methods, multifrequency BIS has been one of the most thoroughly studied techniques; it is a simple, noninvasive, and inexpensive procedure that could be easily used even in children on dialysis. BIS uses the resistance and reactance of electrical current flow through the body to estimate extracellular, intracellular and total body water; lean and adipose tissue mass; and overhydration (OH) [9,11]. Measurement with BIS was shown to correlate well with the deuterated water method, and its use for fluid status adjustment had been associated with increased survival in adult patients on dialysis [12,13]. The association between fluid overload obtained by BIS and cardiovascular morbidity has been well described in various studies [14,15,16]. In children, BIS had been recently reported to be effective, but there has been no correlation between OH and systolic blood pressure (BP) and data on fluid status are limited [17,18,19,20]. Our team previously reported that, in children on HD, intradialytic weight loss correlated well with changes in fluid status measured by BIS, but it did not correlate with the clinical assessment of fluid status [21]. On the other hand, Paglialonga F et al. reported that the use of BIS at their institution improved the left ventricular mass index (LVMI) of children on chronic dialysis and the occurrence of pulmonary edema [22]. Other than these results, evidence on improved outcome of pediatric dialysis after implementing BIS in routine clinical practice had been scarce. The discrepancy between adult and pediatric studies may be explained by differences in body composition and water volumes between adults and growing children [20]. In addition, the impracticality of achieving ideal measurement conditions may reduce the reliability of BIS, especially in younger children [19]. Therefore, the use of BIS in children has been questioned in terms of effectiveness and cautious widespread application because of the low accuracy of the test in children and the absence of a link between fluid overload and clinical parameters [19,20]. Furthermore, most studies on BIS focused on children on HD; therefore, the efficacy of BIS in children on maintenance peritoneal dialysis (PD) remains unclear.

In this study, we evaluated the fluid status by BIS and its association with the clinical characteristics of children on maintenance dialysis. Moreover, we analyzed the clinical outcomes of changing the dialysis prescription in patients with fluid overload based on BIS.

## 2. Methods

### 2.1. Study Design and Data Collection

We retrospectively reviewed the medical records of children receiving maintenance hemodialysis or peritoneal dialysis in Seoul National University Children’s Hospital. All BIS results between January 2017 and December 2019 were recorded. The other data collected were the patient characteristics, including age, height, and weight at the time of BIS; dialysis modality; causes of KFRT; and BP, number of antihypertensive medications, echocardiography results, and the cardiothoracic ratio (CTR) on chest X-ray. The study was approved by the Seoul National University Hospital institutional review board (IRB No. 2006-205-1137). Due to the characteristics of the study, informed consent from the patients was waived.

### 2.2. Performance of BIS

We used the Fresenius body composition monitor (Fresenius Medical Care, Homburg, Germany), which is the most widely used BIS device in children on dialysis [10]. In patients on HD, BIS was performed immediately before the dialysis session. BIS measurement was indicated for routine yearly evaluation or when fluid overload was suspected. Extracellular water (ECW), intracellular water, and total body water volumes were determined, and OH was subsequently calculated using the equations installed in the device [11]. A proportion of OH to ECW (OH/ECW) of >15% was defined as fluid overload, as previously described [2].

### 2.3. Outcomes

The BIS results were evaluated. Fluid status was correlated with the clinical characteristics, such as hypertension, number of antihypertensive medications, and left ventricular hypertrophy (LVH) on echocardiography. Blood pressure was measured using an automatic oscillometric blood monitor during the resting state. Hypertension was collectively defined as systolic and/or diastolic BP above the 95th percentile for age, sex, and height [23,24] or intake of antihypertensive medication to control BP. LVH was defined as left ventricular mass on echocardiography, indexed for height >38.5 g/m^2.7^ [25]. Echocardiography and CTR was obtained between one month before and two months after the BIS measurements. In patients with fluid overload, change in the dialysis prescription according to the BIS finding was confirmed. Clinical assessments of weight reduction, hypertension, number of antihypertensive medications, and subsequent BIS results after change in dialysis prescription were collected. Subsequent BIS was performed at least one month after the previous measurement.

### 2.4. Statistical Analysis

Continuous variables were presented as median values with interquartile range (IQR). The patient demographics and fluid status were compared using Chi-square test or Fisher’s exact test for categorical variables and by the Mann–Whitney U-test for continuous variables. Logistic and linear regression analysis was used to determine the relationship between fluid overload and outcomes, after adjusting for differences in baseline covariates. Values before and after the BIS were compared using Wilcoxon signed rank sum test or McNemar test, as appropriate. Statistical analyses were done by R-project version 4.0.0 (R core team, Vienna, Austria), and a *p* value of < 0.05 was considered statistically significant.

## 3. Results

### 3.1. Patient Characteristics

A total of 49 patients (28 boys, 21 girls) on maintenance dialysis were assessed by BIS at least once during the study period. The median age of the subjects on their first BIS was 13.0 years (IQR, 8.4–16.7 years), and the median time from the initiation of dialysis to the first BIS was 12.2 months (IQR, 0.5–23.3 months). There were 19 patients (38.7%) who were receiving HD as the dialysis modality and 29 patients (59.1%) who had glomerular causes of KFRT.

### 3.2. Fluid Overload Incidence and Association with Clinical Findings

According to BIS, the median value of OH was 0.7 L (IQR, 0.0–1.8 L), and the median OH/ECW was 8.6% (IQR, −0.2–19.2%). Of all patients, 14 (28.5%) had OH/ECW >15% and were considered as having fluid overload (Table 1). In children with fluid overload, the median OH value was 2.9 L (IQR, 1.7–3.8 L) and the median OH/ECW was 24.5% (IQR, 21.2–29.6%). Elevated BP despite the use of antihypertensive medications was significantly more common in patients with fluid overload than in those without fluid overload (78.6% vs. 45.7%, *p* = 0.037). Intake of two or more types of antihypertensive medications was noted in 85.7% of children with fluid overload and in only 48.6% of those without fluid overload (*p* = 0.017). However, fluid overload assessed by BIS was not significantly associated with the cause of CKD, dialysis modality, hypertension, number and types of antihypertensive medications, LVH on echocardiography (available in 31 patients) or CTR on chest X-ray (available in 47 patients) (Table 1). Dialysis duration was longer in patients without fluid overload than in those with fluid overload (*p* = 0.023). Patients with fluid overload tended to have higher of CTR than patients without fluid overload. After adjusting for dialysis duration, BP elevation and the use of two or more antihypertensive drugs remained to be different between the two groups (Table 2).

### 3.3. Change in Hydration Status among Children with Fluid Overload

**Figure 1:**
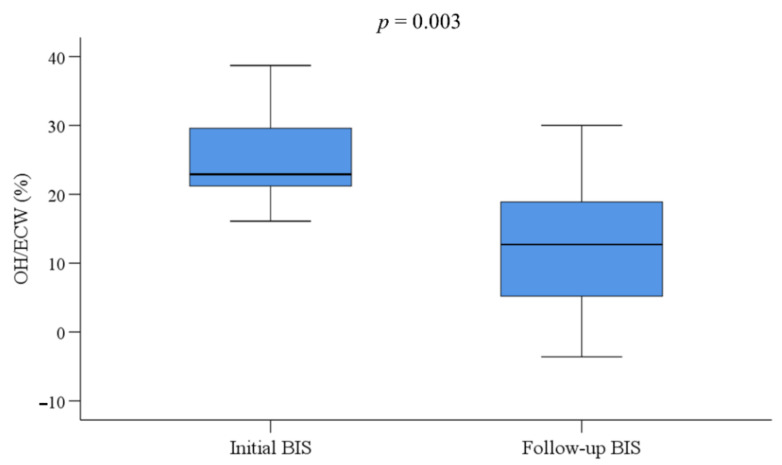
Change in the proportional overhydration to extracellular water (OH/ECW, %) between baseline and follow-up bioimpedance spectroscopy (BIS) (*n* = 13). Statistical analysis was performed using Wilcoxon signed rank sum test.

**Figure 2:**
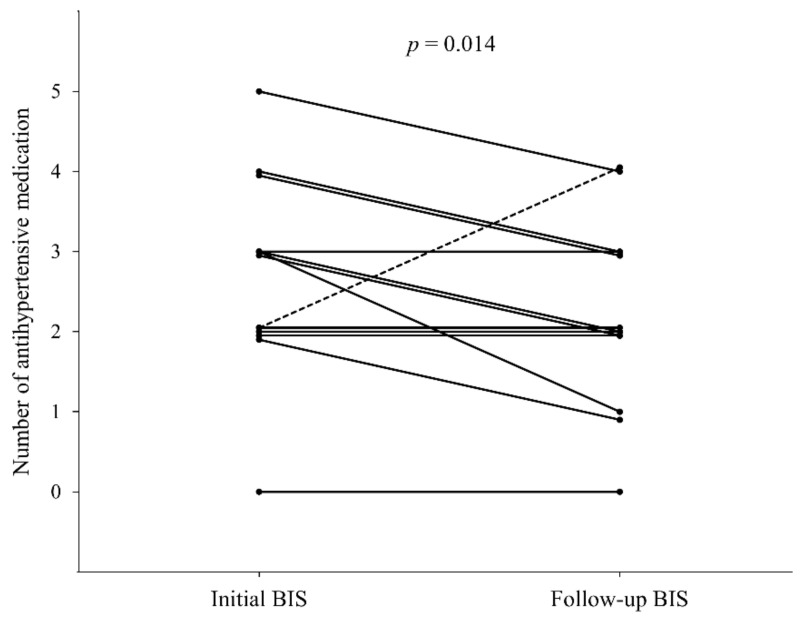
Changes in number of antihypertensive drugs after dialysis prescription in patients with fluid overload (*n* = 13). The dashed line represents a patient on intravenous nicardipine during the initial bioimpedance spectroscopy (BIS) measurement. Statistical analysis was performed using Wilcoxon signed rank sum test in 12 patients except the above case.

Of the 14 children with fluid overload, 13 underwent follow-up BIS after a median of 2.4 months (IQR, 1.7–3.1 months) from the first BIS measurement (Table 3); one patient discontinued PD and received kidney transplantation. Characteristics of these children at initial and follow BIS were shown in Table 4. All 13 children had changes in dialysis prescription; achieved significant weight reduction (*p* = 0.019), from a median of 35.0 kg (IQR, 22.5–49.5 kg) to 32.8 kg (IQR, 21.6–44.3 kg); and had significant reduction of OH/ECW (*p* = 0.003), from a median value of 22.9% (IQR, 21.2–29.6%) to 12.7% (IQR, 5.2–18.9%) (Figure 1). Of the 13 children, 5 were on HD and did not show intradialytic hypotension. After changes in dialysis prescription, the number of antihypertensive medications was reduced in seven children (Figure 2). The number of antihypertensive medications was increased in one patient who was on intravenous nicardipine during the first BIS measurement. Excluding this patient, the median number of antihypertensive medications decreased from 3.0 (IQR, 2.0–3.8) to 2.0 (IQR, 1.5–2.5) after modification of dialysis prescription according to the BIS results (*p* = 0.014). However, hypertension status did not change significantly after the change in dialysis prescription. Follow-up echocardiography was performed on eight patients at a median interval of 9.2 months (IQR, 2.1–13.2 months) and showed a trend of decreased prevalence of LVH and left ventricular (LV) mass. The left ventricular end-diastolic diameter (LVEDD) and CTR significantly decreased at the follow-up exam after the change of dialysis prescription (*p* = 0.049 and *p* = 0.003, respectively).

## 4. Discussion

BIS is becoming common in adult patients because it provides an objective measure of fluid status and its own recommended dry weight. However, the applicability of this method in children has been questionable, because the results of previous studies have not been consistent. In this study, we assessed the clinical relevance of BIS in pediatric dialysis and found that fluid overload assessed by BIS was associated with elevated BP and the requirement of multiple antihypertensive agents in children with KFRT.

In previous studies, the findings of BIS did not correlate well with the clinical assessment of fluid status, in terms of hypertension or LVH on echocardiography [17,18,19,21]. Zaloszyc et al. reported that severe OH by BIS was observed in 11.2% of hemodialysis sessions; however, majority (73%) of that population had normal BP [17]. A prospective study showed that relative OH measured by BIS was associated with peripheral pulse pressure, NT-proBNP, and LVEDD, but not with systolic BP and LVH [18]. In this study, use of two or more antihypertensive drugs and elevated BP were significantly more common in patients with fluid overload than in those without fluid overload. In patients on dialysis, the principal mechanism of hypertension has been thought to be sodium and volume excess, among other factors, such as arterial stiffness, activation of the renin–angiotensin–aldosterone and sympathetic nervous systems, and endothelial dysfunction [26]. Therefore, in general, the requirement for more antihypertensive medications implies excessive volume status in patients on dialysis and, in this study, was correlated with BIS results of fluid overload. However, similar to previous studies [17,18], our study showed that 59.4% of children with elevated BP did not have fluid overload, suggesting that other causes may have contributed to hypertension. 

This study showed that change of dialysis prescription based on the BIS results brought about improvements in both BIS results and the number of antihypertensive drugs. Except one child who was on intravenous antihypertensive medication on the first BIS, none of the children with fluid overload and changed their dialysis prescription increased the number of antihypertensive medications. This finding implied that dry weight estimated by BIS may give useful information to achieve normotension and reduction of antihypertensive medications in pediatric patients on dialysis. Given the rarity of pediatric patients on dialysis, reduction of the number of antihypertensive medications itself may be used as a helpful indicator of the utility of BIS in clinical practice and may foster the routine use of BIS in this population.

We did not find a meaningful correlation between fluid overload according to BIS and LVH. However, our results showed significant decreases of LVEDD and CTR, and a trend toward decreased overall prevalence of LVH and LV mass over time in patients with fluid overload and whose dialysis prescription was modified according to the BIS results. Only 63.2% of the children underwent echocardiography within one month of the BIS measurements; therefore, the study population may have been too small to draw any meaningful conclusion. Pagliolonga et al. reported improvement in echocardiography results after institutional implementation of BIS in children on dialysis. The reported proportion of children with LVH (i.e., LVMI >38.5 g/m^2.7^) was 92.3% in 2007 and 61.1% in 2011 [22]. However, those results were not based on the same subjects and other factors might have affected the results. On the other hand, many studies on adults found a correlation between LVH and fluid overload by BIS [27]. Because echocardiography is relatively costly and does not quantify the volume of fluid overload, BIS can be a more practical and informative method to improve cardiovascular outcome, provided that the agreement between fluid status assessment using BIS and echocardiography findings is verified. Further study that explores sequential changes in echocardiographic results after modification of dialysis prescription in children with fluid overload may be helpful.

Objective assessment of fluid overload is essential and important to optimize the dry weight in patients with dialysis, especially in children, who are vulnerable to the change in volume status. Several noninvasive techniques including BIS have been introduced to clinical practice. The IVC diameter and its collapsing index were reported to be useful to evaluate overhydration in children with kidney failure [28,29,30]. The extravascular lung water assessed by lung ultrasound can be used as an indicator of volume status in children on dialysis [19,31]. Among them, BIS is a simple, rapid, and portable method for measuring fluid distribution, even in children on dialysis. 

There were several limitations in this study. First, it was a retrospective medical review and a single-center study. The results should be interpreted with caution, because not every child on dialysis was assessed using BIS. Therefore, there may have been a bias on the study population inclusion. Implementing BIS as a routine clinical practice for children on dialysis may provide an accurate correlation of BIS with clinical parameters. Second, the relatively short follow-up period was not sufficient to evaluate the long-term efficacy of BIS-based management. Lastly, other cardiovascular indicators, including NT-proBNP, atrial natriuretic peptide, and aortic pulse wave velocity, were not evaluated. Therefore, we may have missed cardiovascular morbidities that were not assessable by echocardiography.

## 5. Conclusions

In this study, we found that BIS was a useful method to assess fluid status in children on dialysis. Fluid overload assessed by BIS was correlated with elevated BP and the requirement of multiple antihypertensive agents. BIS-assisted modification of dialysis prescription improved the clinical parameters and the fluid status itself, which was assessed by BIS. Long-term follow-up and correlation with other objective clinical indicators of fluid overload, such as serum NT-proBNP, would be necessary to assess the clinical relevance of BIS in pediatric dialysis.

## Figures and Tables

**Table 1:** Fluid overload status of bioimpedance spectroscopy and its relevance with clinical characteristics.

	No Fluid Overload (*n* = 35)	Fluid Overload (*n* = 14)	*p* Value
OH/ECW, %	3.1 (−2.3–9.7)	24.5 (21.2–29.6)	<0.001
Male: Female	20:15	8:6	1.000
Age at BIS, years	12.6 (7.0–15.4)	14.2 (10.6–17.2)	0.215
Dialysis duration, months	14.1 (2.6–31.2)	0.8 (0.2–12.2)	0.023
Etiology of KFRT			0.646
Glomerulopathy	20 (57.1)	9 (64.3)	
Nonglomerulopathy	15 (42.8)	5 (35.7)	
Dialysis modality			0.711
Hemodialysis	13 (37.1)	6 (42.9)	
Peritoneal dialysis	22 (62.9)	8 (57.1)	
Hypertension	27 (77.1)	13 (92.9)	0.415
Elevated blood pressure ^a^	16 (45.7)	11 (78.6)	0.037
Number of antihypertensive agents	1 (0.5–3)	2.5 (2–3)	0.124
Two or more antihypertensive drugs	17 (48.6)	12 (85.7)	0.017
Types of antihypertensive agents			
Calcium channel blockers	22 (62.9)	12 (85.7)	0.174
Combined beta- and alpha-blockers	15 (42.9)	9 (64.3)	0.175
RAS blockers	18 (51.4)	6 (42.9)	0.588
Diuretics	2 (5.7)	2 (14.3)	0.568
Vasodilators	2 (5.7)	1 (7.1)	1.000
Echocardiographic parameters ^b^			
LVEDD, cm	4.19 (3.34–4.72)	4.20 (3.84–4.55)	0.633
LVESD, cm	2.70 (2.04–2.93)	2.53 (2.41–2.85)	0.884
Left ventricular mass, g/m^2.7^	49.9 (43.6–68.5)	45.5 (34.9–53.5)	0.147
Left ventricular hypertrophy	18 (85.7)	6 (60.0)	0.172
Cardiothoracic ratio on X-ray ^c^	0.48 (0.42–0.53)	0.53 (0.50–0.56)	0.056

Values are expressed as numbers (%) and median (interquartile range). ^a^ Systolic or diastolic blood pressure ≥95th percentile for age, sex, and height. ^b^ For patients who underwent echocardiography at the time of BIS: no fluid overload group, *n* = 21; fluid overload group, *n* = 10. ^c^ For patients who underwent chest X-ray at the time of BIS: no fluid overload group, *n* = 34; fluid overload group, *n* = 13. BIS, bioimpedance spectroscopy; OH, overhydration; ECW, extracellular water; KFRT, kidney failure with replacement therapy; RAS, renin-angiotensin system; LVEDD, left ventricular end-diastolic diameter; LVESD, left ventricular end-systolic diameter.

**Table 2:** Differences in clinical parameters after adjusting by the duration of dialysis.

**Categorical Variables**	**B**	**Odds Ratio (95% CI)**	***p* Value**
Elevated blood pressure	1.471	4.354 (1.032–18.367)	0.045
Two or more antihypertensive drugs	1.849	6.353 (1.236–32.657)	0.027
Left ventricular hypertrophy	−1.386	0.250 (0.043–1.452)	0.122
**Continuous variables**	**B**	**Standard error (95% CI)**	***p* Value**
Left ventricular mass, g/m^2.7^	−16.244	10.714 (−38.190–5.703)	0.141
Cardiothoracic ratio on X-ray	2.846	1.993 (−1.172–6.863)	0.161

CI, confidence interval. Statistical analysis was performed using logistic and linear regression model for categorical and continuous variables, respectively.

**Table 3:** Changes in the clinical parameters in children with fluid overload after the change of dialysis prescription.

	At the Time of Initial BIS (*n* = 13)	Follow-Up (*n* = 13)	*p* Value
OH/ECW, %	22.9 (21.2–29.6)	12.7 (5.2–18.9)	0.003
Fluid overload	13 (100)	4 (30.7)	0.004
Body weight, kg	35.0 (22.5–49.5)	32.8 (21.6–44.3)	0.019
Hypertension	12 (92.3)	12 (92.3)	1.000
Elevated blood pressure ^a^	10 (76.9)	10 (76.9)	1.000
Number of antihypertensive agents ^b^	3.0 (2.0–3.8)	2.0 (1.5–2.5)	0.014
Two or more antihypertensive drugs	12 (92.3)	10 (76.9)	0.500
Types of antihypertensive agents			
Calcium channel blockers	12 (92.3)	12 (92.3)	1.000
Combined beta- and alpha-blockers	9 (69.2)	8 (61.5)	1.000
RAS blockers	6 (46.2)	7 (53.8)	1.000
Diuretics	2 (15.4)	0	0.500
Vasodilators	1 (7.7)	1 (7.7)	1.000
Echocardiographic parameters ^c^			
LVEDD, cm	4.20 (3.94–4.42)	4.01 (3.67–4.17)	0.049
LVESD, cm	2.53 (2.41–2.79)	2.60 (2.19–2.74)	0.327
Left ventricular mass, g/m^2.7^	45.5 (35.7–60.5)	37.6 (32.5–50.4)	0.161
Left ventricular hypertrophy	5 (62.5)	3 (37.5)	0.625
Cardiothoracic ratio on X-ray ^d^	0.53 (0.48–0.57)	0.48 (0.43–0.51)	0.003

Values are expressed as numbers (%) and median (interquartile range). ^a^ Systolic or diastolic blood pressure ≥95th percentile for age, sex, and height. ^b^ Excluded a case who was on intravenous nicradipine at the time of initial BIS. ^c^ For patients who underwent echocardiography at the time of BIS and after BIS (*n* = 8). ^d^ For patients who underwent chest X-ray at the time of BIS and after BIS (*n* = 12). BIS, bioimpedance spectroscopy; OH, overhydration; ECW, extracellular water; KFRT, kidney failure wigh replacement therapy; RAS, renin-angiotensin system; LVEDD, left ventricular end-diastolic diameter; LVESD, left ventricular end-systolic diameter.

**Table 4:** The change in hydration status and cardiovascular indicators among patients with fluid overload.

Case	Sex/Age (years)	Dialysis Modality	Initial BIS							Follow-Up BIS						
			Bwt (kg)	OH/ECW (%)	SBP/DBP (mmHg)	Number of Antihypertensive Medications	LVM (g/m^2.7^)	LVEDD/LVESD (cm)	CTR	Bwt (kg)	OH/ECW (%)	SBP/DBP (mmHg)	Number of Antihypertensive Medications	LVM (g/m^2.7^)	LVEDD/LVESD (cm)	CTR
1	M/18.0	PD	76.2	26.1	137/83	2	–	–	0.46	68.1	2.0	140/92	1	69.2	5.60/3.48	0.38
2	M/17.1	HD	32.9	31.3	152/83	2	67.4	5.07/3.71	0.53	32.8	30.0	119/82	2	58.0	4.51/3.31	0.49
3	F/14.2	HD	40.4	29.6	110/80	2	33.4	4.28/2.41	0.58	36.6	24.6	110/80	2	37.2	4.15/2.75	0.56
4	M/13.5	HD	20.3	19.2	92/51	0	32.1	3.15/1.77	0.44	21.6	22.5	91/64	0	–	–	0.45
5	M/17.6	PD	62.0	21.5	119/58	3	–	–	–	62.0	12.7	118/62	3	59.5	5.15/3.53	–
6	M/7.5	HD	22.5	22.9	122/79	3	53.6	3.84/2.39	0.52	21.5	3.0	114/73	2	38.0	3.50/2.32	0.52
7	F/8.4	PD	16.5	19.4	133/90	4	–	–	0.58	16.1	18.9	103/70	3	36.5	3.14/2.16	0.48
8	M/12.8	HD	28.0	22.7	122/74	3	42.9	4.04/2.48	0.56	28.7	14.2	115/77	1	31.6	3.58/1.55	0.51
9	F/17.7	PD	58.7	16.1	136/93	2	34.9	4.55/2.85	0.44	57.7	8.2	123/60	2	21.1	3.76/2.05	0.38
10	F/14.9	PD	49.5	38.7	132/60	2 ^a^	–	–	0.53	41.5	5.2	130/87	4	27.5	4.19/2.31	0.43
11	M/6.0	PD	20.9	29.3	113/73	3	68.3	4.12/2.72	0.50	18.5	13.4	110/79	2	42.9	4.10/2.55	0.43
12	F/14.1	PD	46.2	21.2	112/57	5	43.0	4.29/2.58	0.58	44.3	-3.6	120/75	4	33.4	4.18/2.72	0.52
13	F/10.6	PD	35.0	32.8	121/86	4	36.4	3.62/2.41	0.51	30.7	11.2	118/88	2	59.7	3.92/2.65	0.48

^a^ The patient was on intravenous nicardipine during the first BIS measurement. BIS, bioimpedance spectroscopy; Bwt, body weight; OH, overhydration; ECW; extracellular fluid; SBP, systolic blood pressure; DBP, diastolic blood pressure; LVM, left ventricular mass; LVEDD, left ventricle end-diastolic diameter; LVESD, left ventricle end-systolic diameter; CTR, cardiothoracic ratio; M, male; PD, peritoneal dialysis; HD, hemodialysis; F, female.

## Data Availability

Data is contained within the article.
